# Distribution of ticks infesting ruminants and risk factors associated with high tick prevalence in livestock farms in the semi-arid and arid agro-ecological zones of Pakistan

**DOI:** 10.1186/s13071-017-2138-0

**Published:** 2017-04-19

**Authors:** Abdul Rehman, Ard M. Nijhof, Carola Sauter-Louis, Birgit Schauer, Christoph Staubach, Franz J. Conraths

**Affiliations:** 1 Institute for Parasitology and Tropical Veterinary Medicine, Freie Universität, Berlin, Germany; 2 Friedrich-Loeffler-Institute, Institute of Epidemiology, Greifswald, Insel Riems Germany; 3 Department of Epidemiology and Public Health, The University of Veterinary and Animal Sciences, Lahore, Pakistan

**Keywords:** Ticks, Pakistan, Prevalence, Risk factors, Ruminants

## Abstract

**Background:**

Tick infestation is the major problem for animal health that causes substantial economic losses, particularly in tropical and subtropical countries. To better understand the spatial distribution of tick species and risk factors associated with tick prevalence in livestock in Pakistan, ticks were counted and collected from 471 animals, including 179 cattle, 194 buffaloes, 80 goats and 18 sheep, on 108 livestock farms in nine districts, covering both semi-arid and arid agro-ecological zones.

**Results:**

In total, 3,807 ticks representing four species were collected: *Hyalomma anatolicum* (*n* = 3,021), *Rhipicephalus microplus* (*n* = 715), *Hyalomma dromedarii* (*n* = 41) and *Rhipicephalus turanicus* (*n* = 30). The latter species is reported for the first time from the study area. *Rhipicephalus microplus* was the predominant species in the semi-arid zone, whereas *H. anatolicum* was the most abundant species in the arid zone. The overall proportion of tick-infested ruminants was 78.3% (369/471). It was highest in cattle (89.9%), followed by buffaloes (81.4%), goats (60.0%) and sheep (11.1%). The median tick burden significantly differed among animal species and was highest in cattle (median 58), followed by buffaloes (median 38), goats (median 19) and sheep (median 4.5). Female animals had significantly higher tick burdens than males and, in large ruminants, older animals carried more ticks than younger animals. The intensity of infestation was significantly lower in indigenous animals compared to exotic and crossbred cows. Analysis of questionnaire data revealed that the absence of rural poultry, not using any acaricides, traditional rural housing systems and grazing were potential risk factors associated with a higher tick prevalence in livestock farms.

**Conclusion:**

Absence of rural poultry, not performing acaricide treatments, traditional rural housing systems and grazing were important risk factors associated with higher tick prevalence in livestock farms. Age, gender, breed and animal species significantly affected the intensity of tick infestation. This report also describes the presence of *R. turanicus* in the Punjab Province of Pakistan for the first time. The outcomes of this study will be useful in the planning of integrated control strategies for ticks and tick-borne diseases in Pakistan.

**Electronic supplementary material:**

The online version of this article (doi:10.1186/s13071-017-2138-0) contains supplementary material, which is available to authorized users.

## Background

Ticks cause substantial economic losses to resource-poor farming communities, especially in tropical and subtropical regions, where approximately 80% of the world’s cattle population is raised [1]. Tick infestations not only cause direct damage due to the tick bite and blood loss, but ticks can also transmit a wide range of pathogens including zoonotic pathogens, which can produce a serious public health threat [2]. The livestock sector is an integral part of the economy of Pakistan and considered to be the backbone of the rural economy as more than 70% of the population lives in rural areas. Most people depend on keeping livestock for their subsistence [3]. The estimated cattle (*Bos indicus* and *Bos taurus*) population is 41.2 million, and there are 35.6 million buffaloes (*Bubalus bubalis*), 68.4 million goats (*Capra hircus*) and 29.4 million sheep (*Ovis aries*) (figures are based on inter-census growth rate of Livestock Census 1996 & 2006) [4]. Buffaloes are primarily raised in the semi-arid and the arid agro-ecological zones. Cattle and goats can be found throughout the country especially in areas with forage and grazing facilities. A major part of the sheep population is reared in the western and northern hilly areas [5].

Many risk factors are associated with tick infestation in farm animals [6, 7], which in turn has a direct impact on the epidemiology of zoonotic and non-zoonotic tick-borne diseases (TBDs). The effect of environmental factors such as climate [8] and habitat type [9] on tick distribution patterns have been investigated in different parts of the world. Similarly, the effect of host characteristics has conferred various degrees of resistance to tick infestation [10, 11]. Therefore, identification of these risk factors could contribute a vital role in designing cost-effective tick control measures.

So far, many aspects of the epidemiology of ticks and TBDs in Pakistan have not been elucidated. To limit the damage to livestock and humans caused by TBDs, it is necessary to devise an evidence-based tick control program for Pakistan’s livestock sector. To date, only a few studies have investigated the risk factors associated with tick infestation on livestock farms in Pakistan [12, 13].

Although Pakistan’s climatic conditions are suitable for the rapid development of various tick species, there is still a lack of systematic work to investigate the frequency and distribution of tick species infesting ruminants. Many of the previously conducted studies were confined to a small area and did not consider production systems and sampling strategies, which are all factors that can affect prevalence estimates of ticks and TBDs [1]. Moreover, environmental conditions are changing due to global warming, which may alter the distribution patterns and vectorial capacity of ticks [14]. It is crucial to obtain correct and precise information to estimate prevalence and distribution of ticks and tick-borne pathogens, as baseline data for future studies in this field.

The aims of this study, therefore, were to (i) estimate the tick prevalence among farm animals in the semi-arid and arid agro-ecological zone; (ii) identify tick species infesting farm animals in Pakistan; and (iii) identify potential risk factors associated with high tick infestation facilitating the development of effective interventions for tick control.

## Methods

### Study site

**Fig. 1:**
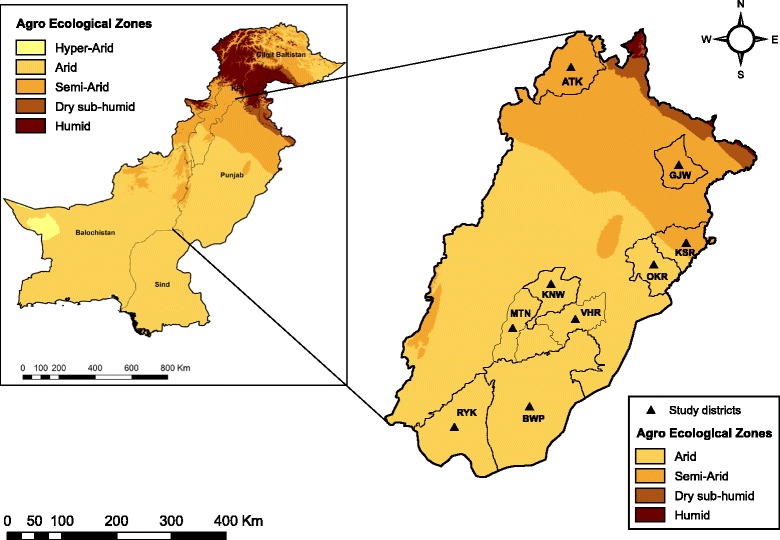
Map of Punjab Province in Pakistan and the districts where tick samples were collected. *Abbreviations*: ATK, Attock; BWP, Bahawalpur; GJW, Gujranwala; KSR, Kasur; KNW, Khanewal; MTN, Multan; OKR, Okara; RYK, Rahim Yar Khan; VHR, Vehari

Pakistan was divided into five agro-ecological zones based on aridity data obtained from the Consultative Group for International Agricultural Research - Consortium for Spatial Information Global-Aridity and Global-PET Database [15]. Of the five agro-ecological zones, two major zones (the semi-arid and arid zone), which cover more than 80% of the country, were selected for sampling. The climate time-series data of the semi-arid and the arid zone are presented in Additional file 1: Table S1. The study focused on Punjab Province (Fig. 1), the most populous region regarding human and animal population. It is located between 28 and 33°N and 70–74°E and shares almost half of the total livestock population in Pakistan, i.e. 18.8 million heads of cattle, 22 million buffaloes, 24 million goats and 7 million sheep [4]. The province has one of the largest irrigation systems in the world with approximately 3,000 irrigation channels. The wet monsoon season from July to September is followed by a retreating monsoon season in October and November with high temperatures countrywide.

### Study design

A cross-sectional study was designed to investigate the distribution of ticks infesting ruminants on livestock farms. The sample size was calculated for large populations assuming a 50% prevalence with 95% confidence level and 10% desired precision, resulting in at least 97 livestock farms to be sampled [16]. This was increased to 108 livestock farms according to the administrative units: in the first stage, 9 (25%) out of 36 districts were selected. Six Union Councils were chosen from each district, and from each Union Council, two villages were selected. In each selected village, one livestock farm was visited for tick sampling and data collection.

The livestock farms were selected in nine districts, namely Attock (ATK), Gujranwala (GJW), Kasur (KSR) in the semi-arid zone and Okara (OKR), Khanewal (KNW), Multan (MTN), Vehari (VHR), Bahawalpur (BWP) and Rahim Yar Khan (RYK) from the arid zone (Fig. 1). All districts are important livestock husbandry regions. Two of them (BWP and RYK) have the highest small ruminant population (1.8 million and 1.5 million, respectively) in Punjab and one (KSR) is home to over 1 million buffaloes. Three of them (KSR, BWP and OKR) are adjacent to India, and two (ATK and RYK) are important livestock trade zones, which connect the northern part of the country with the southern part.

### Collection and preservation of ticks

All tick specimens were collected from September to November 2013 except for MTN district, where the samples were collected in June of the following year. Ticks were collected from buffaloes, cattle, goats and sheep after informed consent from the farm owners had been obtained. To estimate the tick prevalence within livestock herds, two animals from each ruminant species varying in age and sex were randomly selected based on the assumption that at least 50% of the animals on that farm would be infested if ticks are present on the farm [17]. Thus, a minimum of two and a maximum of eight animals were investigated on each farm.

The following predilection sites were examined for the presence of ticks: (i) ears; (ii) brisket (dewlap in the case of cattle); (iii) withers; (iv) knees; (v) udder in the case of females and testes in males along with the perineum region; and (vi) tail [18]. The total tick burden on one side of the animal except for the tail region was estimated according to patch sampling as suggested by Mooring & McKenzie (1995) for obtaining the relative tick burden rather than absolute tick counts [19].

An effort was made to collect a representative proportion of all different types of ticks from predilection sites using a tick removal tool (Ticked Off™, New Hampshire, USA), according to the manufacturer’s instructions. A blunt steel forceps was used for large sized ticks. Ticks were transferred to Safe-Lock Eppendorf® tubes containing 70% ethanol, labelled with a unique sample ID, comprising the farm ID, host species and body location. A separate tube was used for each predilection site. Information regarding each specimen and host related factors including species, breed, gender and age were recorded on predesigned forms.

### Investigation of risk factors

To identify risk factors associated with tick prevalence, a questionnaire containing 21 closed and ten open-ended questions was used. The questionnaire was divided into three parts: (A) farm-related information, (B) tick-related information and (C) herd management-related information. The questionnaire was pilot tested by five farmers in Urdu and Punjabi to ensure that they understood all the questions. The data were collected with the help of local veterinarians.

### Identification of ticks

Tick samples were shipped to the Institute of Parasitology and Tropical Veterinary Medicine, Freie Universität Berlin, Germany for further morphological identification using taxonomic keys covered in Multikey 2.1 software [20] as well as original descriptions and re-descriptions of relevant tick species [21, 22]. The specimens were identified to the species level under a stereomicroscope.

### Molecular identification of ticks

To confirm results of the morphological identification, a fragment of about 750 nt from the second internal transcribed spacer gene (ITS2) of 19 randomly selected tick specimens was amplified and sequenced. Prior to DNA extraction, ticks were washed with distilled water and subsequently homogenised using sterilised pestles in 1.5 ml Safe-Lock Eppendorf® tubes. DNA extraction was performed using the NucleoSpin® Tissue Kit (Macherey-Nagel GmbH & Co. KG, Düren, Germany), according to the manufacturer’s recommendations for the purification of genomic DNA from insects. Purified DNA was quantified using an Epoch Spectrophotometer (BioTek Instruments, Bad Friedrichshall, Germany). The following primers were used: Metastriata ITS-F (5′-AGG ACA CAC TGA GCA CTG ATT C-3′) and Metastriata ITS-R (5′-ACT GCG AAG CAC TTR GAC CG-3′). Polymerase chain reaction (PCR) amplification was performed in a total reaction volume of 25 μl containing 2.5 μl of 10× Maxima Hot Start Taq Buffer, 2.5 μl of 1.5 mM MgCl_2_, 2.5 μl of 2 mM dNTPs, 1 μl (10 μM) of each primer, 0.25 μl (2U/μl) of Maxima™ Hot Start Taq DNA Polymerase (Thermo Scientific, Karlsruhe, Germany) and 2.5 μl of template DNA in a C1000™ Thermal Cycler (Bio-Rad Laboratories, Munich, Germany). The cycling conditions comprised a 5 min denaturation and polymerase activation step at 95 °C, 40 cycles of 95 °C for 30 s, 57 °C for 30 s and 72 °C for 50 s and a final extension step for 5 min at 72 °C. A negative control was used to authenticate the PCR reaction. PCR amplicons were visualised by gel electrophoresis, purified using the Zymoclean™ Gel DNA Recovery Kit (Zymo Research Corporation, Freiburg, Germany) according to the manufacturer’s instructions and sequenced (LGC Genomics, Berlin, Germany). The resulting sequences were subjected to a Basic Local Alignment Search Tool (BLAST) analysis (www.ncbi.nlm.nih.gov/blast/). Following confirmation of tick species identity by the ITS2 gene, three *R. microplus* specimens (one female and one male from buffalo, one female from cattle) were further characterised as it was known that different clades exist in this species. A 1,592 base pair fragment of the cytochrome c oxidase subunit 1 (*cox*1) gene from these three *R. microplus* specimens were amplified and sequenced using primers Rhcox1 F (5′-CCG CCT AAA CTT CAG CCA TT-3′) and Rhcox1-R (5′-GTC TGA AAA TG(C T)TA ATT GAG ATC AAG-3′) with identical PCR conditions except for the extension time, i.e. 100 s, as described for the amplification of the ITS2 gene.

### Creation of the aridity map

The Global-Aridity dataset was downloaded as one grid layer representing the annual average for years 1950–2000 (http://www.cgiar-csi.org/data/global-aridity-and-pet-database). The Aridity Index (AI) values reported within the Global-Aridity dataset had been multiplied by a factor of 10,000 to derive and distribute the data as integers (with four decimal accuracy). This multiplier had been used to increase the precision of the variable values without using decimals (real or floating values are less efficient regarding computing time and space compared to integer values). Therefore, Global-Aridity values were divided by 10,000 to retrieve the values in the correct units. The data relevant to Pakistan was extracted by a mask of a polygon indicating the borders of Pakistan (Pakistan Admin). The raster map (PakAridityMap) was converted to a polygon. Subsequently, the aridity polygon features for Punjab Province (PunjabAridity) were extracted from “PakAridityMap”. A higher AI and darker colours represent more humid conditions, while low AI Index and lighter colours represent higher aridity.

### Statistical analyses

Tick prevalence on the animal level was calculated as the number of animals infested with ≥ 1 ticks, divided by the total number of animals examined. For the herd prevalence, a farm was considered positive, if at least one animal on the farm was found to carry ticks during the farm visit. The binomial confidence intervals (CI) for proportions were estimated in R using the package ‘*binom*’ [23] with the ‘exact-Clopper-Pearson interval’ method. The prevalence of ticks in agro-ecological zones was compared using the Fisher's exact test for count data. Tick burdens in animals in different agro-ecological zones were compared using the Kruskal-Wallis test. To assess the effects of host traits (e.g. gender, breed and species) on tick burden, the Mann-Whitney-Wilcoxon test with continuity correction and the Kruskal-Wallis test were used. The Tukey and Kramer (Nemenyi) test with the Tukey-distribution approximation for independent samples was applied in the *post-hoc* analysis to test for the effect of animal species and cattle breeds on tick burden. The association of age and tick burden was evaluated using Spearman’s rank correlation.

### Risk factors study

To measure the effect of various determinants on tick prevalence, a multivariable logistic regression model was built. In the first step, a univariable analysis (Fisher’s exact test) was performed to select the variables (predictors) with *P* < 0.2, which were included with an additive mode of interaction in the multivariable model [24]. Subsequently, the variables were removed from the multivariable model one by one using a backwards stepwise selection approach, if they were not significant and not a confounder. A variable was considered statistically significant if the *P*-value for that specific variable was less than 0.05. Potential confounding effects were evaluated by assessing the change in any remaining parameter estimate. If this change was greater than 20% as compared to the full model, the variable was considered a confounder. Farms were categorised at a dichotomous level (low tick prevalence: 0; high tick prevalence: 1, using a cut-off at 80% prevalence). The ultimate model was fitted with the farm category (low or high infested) as the response variable. Deviance residuals were examined for homoscedasticity and normal distribution. Pearson goodness-of-fit statistic (*χ* ^2^) was applied to assess model fit. Additionally, Akaike information criterion (AIC) values were also utilised to assess the quality of the model. The multivariable model was run using the glm() function. The link function “logit” was used in the model to report the coefficient, the ratio of the coefficient to its standard error and the *P*-value. Odds ratios (OR) along with 95% CI were calculated using the exp() function. The software records an OR of 1 for the reference variable. When the upper limit of the CI of the OR of the examined variable was below than 1, it was considered as a protective factor, and if the lower limit of the CI was above 1, it was considered as a risk factor. OR are presented for the independent variables that showed statistical significance in the multivariable analysis. All statistical analyses were performed with R for Windows software (version 3.2.1., http://www.r-project.org/) and RStudio as an interface (version 0.99.447, Inc., Boston, MA, USA, https://www.rstudio.com/).

Maps were produced in the ArcMap software environment version 10.3 (Esri, Redlands, Ca, USA). The base map for Pakistan was obtained from the database of Global Administrative Areas and was set for Gujrat, Gujranwala, Narowal and Okara districts using the ‘dissolved function’.

## Results

### Demographic characteristics of the study population

A total of 471 ruminants (194 buffaloes, 179 cattle, 80 goats and 18 sheep) were examined on 108 livestock farms in two different agro-ecological zones: semi-arid zone (*n* = 139) and arid zone (*n* = 332). The majority (66%) of the animals were female. The median herd size was 10 (Q1-Q3: 8–15) animals. The median age of the animals was 2.5 years (Q1-Q3: 1.0–4.5). The median age in large ruminants was 3.0 years (buffalo = 3.1 and cattle = 3.0) and 1.5 years in small ruminants. The median age of the infested animals was 3.0 years (Q1-Q3: 1.0–5.0) while it was 1.5 years (Q1-Q3: 1.1–2.5) in the non-infested group.

### Tick species

In total, 3,807 ixodid ticks (female: 1,303; male: 1,261; nymph: 1,231; larva: 12) were collected from 108 livestock farms (Table 1). They belonged to four species. *Hyalomma anatolicum* (*n* = 3,021, 79.3%) was the most common species, followed by *R. microplus* (*n* = 715, 18.8%), *H. dromedarii* (*n* = 41, 1.1%), and *R. turanicus* (*n* = 30, 0.8%). *Rhipicephalus microplus* was predominant in the semi-arid zone, while *H. anatolicum* was the most common tick species in the arid zone. *Hyalomma dromedarii* and *R. turanicus* were only present in the arid zone. *Hyalomma anatolicum* was found in all the districts of the province, while *R. microplus* was absent in MTN, BWP, and RYK. In all the districts, multiple tick species were found except in MTN district, where only *H. anatolicum* was detected. *Rhipicephalus microplus* and *H. dromedarii* were mainly found in cattle, whereas *R. turanicus* was mainly found on goats. *Hyalomma anatolicum* infested all ruminant species.

**Table 1:** Distribution of tick species and their associated host animal species in the semi-arid and the arid agro-ecological zones of Pakistan

Districts	Host	*Hyalomma* spp.		*Rhipicephalus* spp.		Tick species (%)
		*H. anatolicum* ^*b*^N/M/F^*c*^	*H. dromedarii* N/M/F	*R. microplus* N/M/F	*R. turanicus* N/M/F	
Semi-arid						
ATK	Buffalo	–	–	0/5/16	–	*H. anatolicum* = 35.9 *R. microplus* = 64.1
	Cattle	0/25/31	–	10/48/78	–	
	Goat	–	–	0/10/10	–	
	Sheep	6/24/24	–	–	–	
GJW	Buffalo	0/0/3	–	1/33/105	–	
	Cattle	0/7/11	–	7/50/94	–	
	Goat	–	–	0/8/12	–	
	Sheep	–	–	–	–	
KSR	Buffalo	0/55/49	–	9/7/30	–	
	Cattle	7/53/52	–	19/15/50	–	
	Goat	3/0/0	–	3/0/3	–	
	Sheep	–	–	3/0/0	–	
Arid						
OKR	Buffalo	20/140/84	–	7/1/16	–	*H. anatolicum* = 94.3 *H. dromedarii* = 1.5 *R. microplus* = 3.1 *R. turanicus* = 1.1
	Cattle	23/115/65	–	8/1/25	–	
	Goat	–	–	–	–	
	Sheep	–	–	–	–	
KNW	Buffalo	72/59/35	–	4/1/4	–	
	Cattle	214/55/27	–	0/2/5	–	
	Goat	21/0/0	–	–	–	
MTN^a^	Buffalo	65/66/96	–	–	–	
	Cattle	168/110/104	–	–	–	
	Goat	26/4/2	–	–	–	
VHR	Buffalo	83/52/47	–		–	
	Cattle	85/39/26	–	0/5/10	–	
	Goat	43/12/4	–	–	–	
	Sheep	0/0/2	–	–	–	
BWP	Buffalo	57/65/41	0/4/1	–	0/4/3	
	Cattle	24/25/22	13/16/6	–	–	
	Goat	35/15/9	0/1/0	–	6/0/2	
RYK	Buffalo	71/86/59	–	–	–	
	Cattle	84/31/30	–	–	–	
	Goat	20/12/9	–	–	14/0/1	
	Sheep	–	–	–	–	
Total	3807	1127/1050/832	13/21/7	71/186/458	20/4/6	
Mean % (95% CI)		79.3 (78.0–80.6)	1.1 (7.7–1.4)	18.8 (17.5–20.0)	0.8 (0.5–1.1)	

*Abbreviations*: *AEZ*, Agro-ecological zone, *CI* Confidence interval

^**a**^In MTN (Multan district) the samples were collected at a different time (following year, June) and only *H. anatolicum* species was found

^**b**^Larvae (all belong to *H. anatolicum*) are not presented in the table (MTN = 5; VHR = 3; BWP = 3; RYK = 1)

^c^N/M/F: Nymphs/Males/Females

The morphological identification was supported by sequencing a partial fragment of the ITS2 gene from 19 randomly selected samples followed by a BLAST analysis. Of these 19 sequences, 15 samples previously identified as *H. anatolicum* showed 100% identity to *H. anatolicum* strains from China (HQ005303) and Iran (FJ593703). Two *R. microplus* samples had 100% identity to a *R. microplus* isolate from Laos (KC503276). The sequence of *H. dromedarii* showed 99% identity to a registered sequence of *H. dromedarii* from a dromedary camel in India (JQ733570), whereas the sequence of *R. turanicus* was 99% identical to the *R. turanicus* isolate 80-T-He4 (accession no. KF958417). In addition, the BLAST results of the *cox*1 gene sequences from three *R. microplus* ticks revealed the highest identity (95.5%) with a Chinese *R. microplus* isolate from Guizhou, China (KC503259). Representative ITS2 sequences for each tick species and *cox*1 (only for *R. microplus*) were submitted to GenBank and under the following accession numbers: *H. anatolicum* (KY373255, KY373256), *R. microplus* (ITS2: KY373257, *cox*1: KY373260), *H. dromedarii* (KY373258), *R. turanicus* (KY373259).

### Tick prevalence

All livestock herds, irrespective of their geographic location, were found infested with one or multiple tick species. Within the herds, the tick prevalence varied from 20 to 100% (Mean ± SD; 80 ± 20%). The overall proportion of tick-infested ruminants was 78.3% (369/471); this was highest in cattle (89.9%), followed by buffaloes (81.4%), goats (60.0%) and sheep (11.1%) (Table 2). The tick prevalence was significantly lower in the semi-arid zone as compared to the arid zone. Out of all infested animals, 71.0% (*n* = 265) were infested with *H. anatolicum*, 17.0% (*n* = 59) with *R. microplus*, 1.4% (*n* = 5) with *H. dromedarii*, 1.1% (*n* = 4) with *R. turanicus* and 9.5% (*n* = 36) were found to have a mixed infestation with more than one tick species.

**Table 2:** Cumulative tick burden, prevalence and median tick burden in ruminants on livestock farms in the context of agro-ecological zones and districts of Punjab Province, Pakistan. Prevalence (OR = 0.60, 95% CI: 0.37–0.98, *P* = 0.037) and tick burden (*W* = 10,650, *P* = 0.002) were significantly different between the agro-ecological zones

AEZ Districts	NAI/NAO/NTC	Animal species (NAI/NAO)				Tick burden per animal^a^	Prevalence in % (95% CI)
		Buffalo	Cattle	Goat	Sheep		
Semi-arid	100/139/976	42/62	47/56	10/18	1/3	36 (14–66)	72 (64–79)
ATK	30/43/290	10/17	16/17	04/08	0/1	24 (11–71)	70 (54–83)
GJW	32/47/328	16/21	12/19	04/06	0/1	34 (12–63)	68 (33–81)
KSR	38/49/358	16/24	19/20	02/04	1/1	37 (27–73)	78 (63–88)
Arid	269/332/2,831	116/132	114/123	38/62	1/15	46 (30–67)	81 (76–85)
OKR	39/55/505	22/24	17/17	00/06	0/8	41 (32–54)	71 (57–82)
KNW	46/51/499	20/22	22/23	04/06	0/0	48 (29–86)	90 (78–97)
MTN^b^	45/51/646	18/20	23/24	04/07	0/0	82 (56–110)	88 (76–96)
VHR	45/58/411	14/20	20/22	10/12	1/4	43 (25–63)	78 (65–87)
BWP	48/57/352	20/22	17/20	11/15	0	34 (27–48)	84 (72–92)
RYK	46/60/418	22/24	15/17	9/16	0/3	42 (28–57)	77 (64–87)
Total	369/471/3,807	158/194	161/179	48/80	2/18	43 (27–67)	78 (74–82)

*Abbreviations*: *AEZ* Agro-ecological zone, *NAI* Number of animal infested, *NAO* Number of animal observed, *NTC* Number of ticks collected, *ATK* Attock, *GJW* Gujranwala, *KSR* Kasur, *OKR* Okara, *KNW* Khanewal, *MTN* Multan, *VHR* Vehari, *BWP* Bahawalpur, *RYK* Rahim Yar Khan, *CI* Confidence interval

^a^Values for tick burden are presented as median (1st and 3rd quartiles)

^b^Tick samples were collected at a different time (June 2014)

### Tick burden

**Fig. 2:**
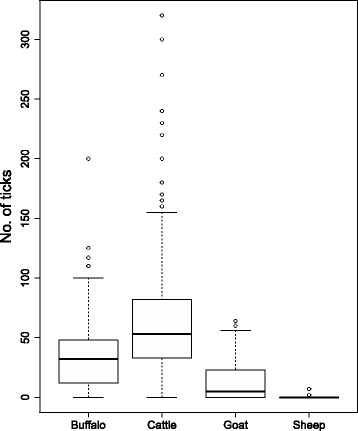
Box-and-whisker plots for the tick burden recorded in different animal species

The median of the recorded tick burden (43 ticks per animal, Q1-Q3: 27–67) was significantly different among the examined animal species (*χ* ^2^ = 115.42, *df* = 3, *P* < 0.001). It was highest in cattle (median 58), followed by buffaloes (median 38), goats (median 19) and sheep (median 4.5) (Fig. 2). Large ruminants were more heavily infested than small ruminants (*P* < 0.001), and between bovines, the infestation was higher in cattle (*P* < 0.001) than in buffaloes. Tick burden on livestock farms was significantly lower (*W* = 738, *P* < 0.001) in the semi-arid zone as compared to the arid-zone (Table 2).

### Effect of host characteristics on tick burden

**Fig. 3:**
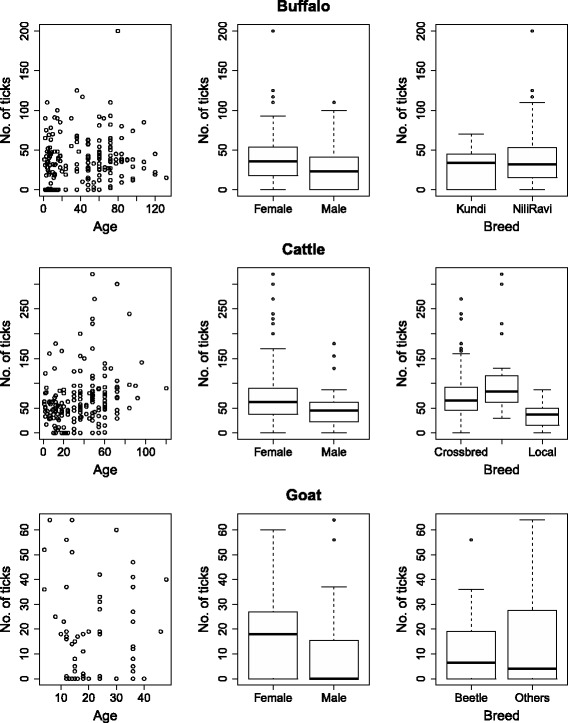
Intensity of infestation in animals in relation to their age (in months), gender and breed

In large ruminants, older animals carried more ticks than younger animals (Table 3). It was also observed that female animals had higher tick burdens than male animals. The intensity of infestation was significantly different among cattle breeds (*χ* ^2^ = 55.42, *df* = 2, *P* < 0.001), where indigenous animals had lower tick burdens as compared to exotic (*P* < 0.001) and crossbred cows (*P* < 0.001), while the difference was not statistically significant between crossbred and exotic cattle (*P* = 0.11) (Fig. 3). In other ruminant species, a statistically significant effect of breed on tick burden could not be demonstrated.

**Table 3:** Effect of host characteristics on tick burden in livestock

Variable	Statistics	Buffalo	Cattle	Goat	Sheep	Overall
Age	*P-*value	0.02	< 0.001	0.6806	0.988	< 0.001
	Spearman’s rho	0.167	0.27	-0.047	-0.003	0.215
Gender	*P-*value	0.002	< 0.001	0.014	0.04	< 0.001
	95% CI	3.00–22.99	7.99–33.00	< 0.01–10.69	< 0.01–10.7	10.99–24.99
	Wilcoxon-statistic	4,643	4,599	1,047	48	32,772
Breed	*P-*value	0.204	< 0.001	0.628	0.935	na
	95% CI	-18.99–1.99	na	-6.0–1.99	< -0.01–0.02	na
	Wilcoxon-statistic	2,284	55.42^a^	640	40.5	na

*Abbreviations*: *NA* Not applicable, *CI* Confidence interval

^a^Kruskal-Wallis *χ* ^2^ value

### Tick infestation and control

Only a small proportion of farms (11.1%, 95% CI: 5.9–18.6) raised a single ruminant species, while the majority (88.9%, 95% CI: 81.4–94.1) held more than one species. Cattle were present on 87.0% (95% CI: 79.2–92.7) farms, buffaloes on 92.6% (95% CI: 85.9–96.8), goats on 39.8% (95% CI: 30.5–49.7) and sheep on 10.2% (95% CI: 5.2–17.5). Additional file 2: Tables S2 and S3 show the results of the descriptive analysis for the categorical and numeric variables.

When farm owners were asked about the months when ticks occur on their farm, most (64.8%, 95% CI: 55.0–73.8) reported the period from March to November, while 15.7% (95% CI: 9.4–24.0) observed ticks throughout the year, and the remaining 19.4% (95% CI: 12.5–28.2) did not know. Regarding the months of highest tick infestation, 83.3% (95% CI: 74.9–89.8) of the farmers reported the period from June to September and 16.7% (95% CI: 10.2–25.1) did not know.

Almost one-third of the total farmers (29.0%, 95% CI: 20.6–38.5) used acaricides to control ticks. When they were asked about the names of the acaricides used, 16 (51.6%, 95% CI: 33.1–69.8) out of 31 farmers did not know, whereas the remaining farmers (48.4%, 95% CI: 30.2–66.9) answered that they used ivermectin (injectable) (19.4%), trichlorfon 97.1% (topical) (12.9%) or both (16.1%). Most the farmers (81.6%, 95% CI: 71.0–89.5) used at least one tick control method like acaricides, hand picking, keeping rural poultry, topical application of “Taramira oil” (*Eruca sativa*) on the body of the animals, while only a few farmers (18.4%, 95% CI: 10.5–29.0) stated that they had not used anything against ticks. It was a common practice that farmers (95.4%, 95% CI: 89.5–98.5) offered only green roughages to their animals, except for milking animals. Only five farmers (4.6%, 95% CI: 1.5–10.5) fed all their animals with green roughages plus concentrate (energy-rich feed).

Almost half of the farmers (47.2%, 95% CI: 37.5–57.1) reported a behavioural change in tick-infested animals, e.g. restlessness and scratching. More than half of the farmers (58.3%, 95% CI: 48.5–67.7) had noticed a drop-in milk production of the tick-infested animals, while the others had not observed a change in performance (13.0%, 95% CI: 7.3–20.8) or did not know (28.7% 95% CI: 20.4–38.2). Only two farmers reported visits of para-veterinary or veterinary staff daily, while the others reported that these staff visited the farm only when animals became sick (*n* = 106) or when artificial insemination (*n* = 65) or vaccination (*n* = 24) was needed.

### Variables included in the multivariable logistic regression model

The initial main effect model included eight variables, which had yielded *P* < 0.2 in univariable analysis (see Additional file 3: Tables S4) (AIC: 109.2). The final logistic regression model is presented in Table 4 (AIC: 102.1). The Pearson goodness-of-fit statistic showed that the model adequately fitted the data (*P* = 0.748). The presence of rural poultry on farm significantly (*P* = 0.006) affected the tick prevalence, and the odds of having higher tick prevalence on farms where rural poultry was absent were 4.4 times as high as on farms with rural poultry. The tick prevalence was significantly lower (*P* < 0.001) on farms where acaricides had been used. The housing type also had a significant effect on the tick prevalence (*P* = 0.007), and the chances of getting higher tick prevalence on farms with traditional rural housing system were almost 13 times as high as on farms with open houses. The feeding method was also an important variable associated with the tick prevalence and farms, where grazing was practised, had a higher prevalence (*P* = 0.003) as compared to farms with a stall-feeding system.

**Table 4:** Summary of variables included in the final multivariable logistic regression model. AIC: 102.1 as compared to 109.2 for the preliminary main effect model. Pearson goodness-of-fit: *P* = 0.748

Variable	Response categories	Coefficient	Odds ratio	95% CI	*P-*value
Housing type	Open		1		
	Traditional rural	2.6	13.1	2.4–118.0	0.007
Feeding method	Stall feeding		1		
	Grazing	2.5	12.6	2.9–96.4	0.003
Use of acaricide(s)	Yes		1		
	No	2.0	7.5	2.4–26.7	< 0.001
Rural poultry	Present		1		
	Absent	1.5	4.4	1.6–13.0	0.006

*Abbreviation*: *CI* Confidence interval

## Discussion

### Description of identified tick species

To our knowledge, this is the first report from Pakistan in which the identified tick species are confirmed using molecular techniques, which can be utilised to identify and discriminate different species and subspecies of ticks in cases where the morphological identification is doubtful or ambiguous [25]. Previous studies from the country were only based on morphological identification [6, 26, 27], and most of them identified tick samples at the genus level [28–30].

Overall, *H. anatolicum* was the most abundant tick species found in this study, which agrees with previous studies from Pakistan [26, 31] and bordering countries as well [32, 33]. It parasitized all the animal species in both the agro-ecological zones. *Hyalomma anatolicum* is a potential vector responsible for the transmission of *Theileria annulata* and *Theileria lestoquardi* in Pakistan [26]. Furthermore, it can cause serious damage to cattle hides because of its long mouthparts. Notably, these ticks preferentially feed on the udder and teats of cattle [34] and may cause serious problems in the suckling of calves.

*Rhipicephalus microplus* was the second most prevalent tick species, which also infested all animal species in both the agro-ecological zones. Our finding that *R. microplus* was the dominant tick species in the semi-arid zone (northern part of the province), is in accordance with those of a local study conducted on two governmental farms [28], where the authors noticed a clear preponderance of *R. microplus*. The species was absent in the southern part of the province (MTN, BWP and RYK), which is a drier region. The geographical distribution and abundance of this tick species have been greatly promoted by the retaining water capacity of the underlying layer of the soil and the increased relative humidity. It was recently reported that *R. microplus* is, in fact, a species complex, which consists of at least five taxa: *R. annulatus*, *R. australis*, *R. microplus* clade A, *R. microplus* clade B and *R. microplus* clade C [35, 36]. The Punjabi *R. microplus,* whose *cox*1 gene we sequenced, showed the highest identity to a *R. microplus cox*1 sequence from Malaysia (KM246868), referred to as haplotype 17 in [36], suggesting that it belongs to clade C of the *R. microplus* complex, which also comprises ticks from India and Malaysia.

The ecological niche for *H. dromedarii* was confined to the BWP district in the arid zone, from where it has already been reported, but always in small numbers [37]. The major part of this district consists of desert, where the camel production is common. It is important to note that *H. dromedarii* is specialised to feed on camels. Moreover, the finding that the presence of *H. dromedarii* appeared to be confined to this district is perhaps explicable by the influence of the low relative humidity in the area on this desert-adapted tick species [37].

Here we report for the first time the occurrence of *R. turanicus* in Punjab Province. Only limited numbers (*n* = 30) were found, and the tick was confined to the BWP and RYK districts of the arid zone. The tick species was only found on water buffaloes and goats. Although some studies have been conducted in the past, *R. turanicus* has never been reported from Punjab Province; however, it has been reported from Sindh Province, but quite a long time ago [37]. McCarthy [38] also found *R. turanicus* in Pakistan, but the author considered it as a subspecies of *R. sanguineus* due to the previous work of Pervomaisky [38, 39]. Nevertheless, it has been identified on domestic ruminants in neighbouring countries, namely Iran [32], India [33], Bangladesh [40] and China [41]. A report from Europe concluded that *Rhipicephalus* sp. III from Pakistan and India were morphologically and genetically similar to, but still different from, *R. turanicus* [42]. After considering these reports, we conclude that *R. turanicus* has been there all the time but was misreported as *R. sanguineus*, as the two are morphologically very difficult to distinguish and molecular techniques were previously not utilised for confirmation.

These tick species infest a broad range of host species and transmit several important pathogens including viruses, bacteria and protozoa of medical and veterinary importance. *Hyalomma anatolicum* has been reported as the principal tick vector for Crimean-Congo haemorrhagic fever (CCHF) [43]. After several outbreaks of CCHF in Pakistan, the infection has now become an endemic problem [44] and the possibility of transmission of CCHF virus to farmers, especially when hand picking is common practice, cannot be neglected [45]. *Rhipicephalus microplus* and *Hyalomma* spp. are potential vectors of *Anaplasma centrale* and *A. marginale* [1], the causative agents of bovine anaplasmosis in Pakistan [46]. Furthermore, these tick species have been reported to transmit different protozoan parasites, including *Theileria annulata* and *Babesia bovis* in buffalo and cattle in Pakistan [1, 26]. Although scanty, the presence of *R. turanicus* may still be of medical as well as veterinary importance as it is known to harbour *Coxiella burnetii*, the causative agent of Q fever, which has recently been recorded in Pakistan [47].

### Tick prevalence in animal species

It is evident from the results that the tick prevalence significantly differed between animal hosts, which concurs with previous studies [40, 48]. The observed higher tick prevalence in cattle as compared to buffaloes might be linked with the drier habitats and thinner skin of cattle as compared to the marshy habitats and thicker skin of buffaloes [12], and host genetics may also play an important role [49].

Limited information is available about tick prevalence in small ruminants in Pakistan, as only a few studies have focused on these animals. In general, the tick prevalence observed in the present study was higher in goats (60.0%, 95% CI: 48.4–70.8) as compared to sheep (11.1%, 95% CI: 1.4–34.8), which is also in agreement with a local study [48]. Although a reason for lower tick prevalence in sheep is not evident, one could speculate that the hairy wool might be an important protective factor against tick infestation [48]. However, the prevalence estimate in sheep cannot be compared and extrapolated because of the small number of animals in our study. Other studies observed a similar (60.1% %, 95% CI: 56.6–63.5) [6] or slightly lower tick prevalence of 41.5% (95% CI: 36.9–46.3) [28]. However, the latter study was conducted in the northern part of the province, where we also observed a lower prevalence.

### Tick prevalence in agro-ecological zones

The present study revealed that all the livestock herds were found infested with one or multiple tick species. Within-herd tick prevalence ranged from 20 to 100% (Mean ± SD:  80 ± 20%). The tick prevalence on an animal level and median tick burden were lower in the semi-arid zone as compared to the arid zone. The semi-arid zone is located at a higher elevation and observes low annual mean temperatures (minimum and maximum) as compared to the arid zone. Previous reports from Pakistan did not consider the agro-ecological zones and were based on administrative units only [1]. Nevertheless, fluctuations in tick prevalence have been reported in different areas of the same region [13]. A study from the lower Punjab reported a significant difference in tick prevalence between the two districts and even among different areas of the same district [12]. Moreover, variations in tick prevalence in buffaloes of different geographical conditions have previously been documented in Pakistan [12]. However, the variations in tick prevalence within the same geographical region can be attributed to differences in husbandry practices including tick control strategies and awareness of the farmers [40].

### Tick burden

Like the tick prevalence, tick burden (also known as the intensity of tick infestation) was also highest in cattle followed by buffaloes, goats and sheep. Our results agree with a previous study from Pakistan [26]. These authors reported a higher intensity of tick infestation in cattle as compared to buffaloes, but they did not mention the average tick burden. In the past, studies have shown that buffaloes were a less suitable host than cattle for *R. microplus* ticks, the second most common tick species in our samples. A plausible explanation could be that the thick skin of the buffalo reduced the ability of these ticks to attach because of their short hypostome. Additionally, the immune system of the buffalo showed an increased sensitivity to tick proteins than that of cattle [50].

### Effect of host characteristics on tick burden

#### Gender

Our finding that tick burdens were higher in female animals as compared to males is consistent with previous studies carried out in ruminants [13, 14]. Male animals are mainly used for draught and breeding purposes throughout the year, and for the sacrificial purpose on the celebration of “Eid-ul-Adha”, the biggest sacrifice and holy festival of Muslims. Therefore, they receive more attention, like frequent grooming including the manual removal of ticks, which would result in low tick burdens. A few weeks before the festival, animals, particularly goat and sheep, are transported for sale from the farms to animal markets in the big cities. It is pertinent to mention that the sacrifice festival held during the field work, so this could explain the low number of sheep and goats sampled. It has been postulated that both, pregnancy and lactation stress, decrease the resistance in females [51].

#### Age

A significantly lower tick burden in calves as compared to older animals agrees with previous studies [52]. A previous study estimated that adult cattle had a higher chance of carrying ticks (OR = 12.3) than calves [53]. The lower tick burdens recorded in calves could be due to a combination of factors, including the frequent grooming of calves, especially head, ears and neck regions, by their dams and the smaller surface area of younger animals as compared to adults [54]. Furthermore, young animals seem to be more capable of protecting themselves from ticks by innate and cell-mediated immunity [55], although it must be stressed that we did not evaluate the immune status of the animals in our study.

#### Breed

Our results showed that tick infestation was highest in exotic cattle (Taurine cattle or *Bos taurus taurus*) followed by crossbred and indigenous cows (Zebu cattle or *Bos taurus indicus*), which is in line with previous reports from Pakistan [12, 27], in which a similar pattern among cattle breeds was reported. Higher tick infestation in exotic breeds as compared to indigenous cattle has also been reported in Argentina [56], Ethiopia [57] and Egypt [14]. Resistance to one-host ticks, e.g. *R. microplus* is related to the proportion of zebu genes in the breed [58]. In *Bos indicus* and their crosses, the resistance against different tick species has been found a highly heritable trait [59]. Although the mechanism of resistance acquired by the indigenous breeds is not fully understood, it could be related to pre-immunity against ectoparasites, which often established through frequent contacts with the parasites at an early stage of life [27]. Differences in the immune responses among cattle breeds have been observed, which might play an important role in the development of tick resistance [60]. Moreover, histamine stimulated grooming by the host is responsible for tick removal, and higher concentrations of histamine are measured in cattle that are resistant to ticks as compared to non-resistant cattle [61]. However, plausible factors, which affect the breed susceptibility for tick infestation still need to be explored in indigenous cattle breeds of Pakistan.

Keeping the above facts in mind, host-tick resistance can be exploited in cattle breeding programmes, which could contribute to the biological control of tick infestation. However, an often-heard drawback of selective breeding for tick resistance is that resistant breeds typically have poor production characteristics (growth, milk yield) compared to European breeds (which have been selected for a high production).

### Risk factors associated with higher tick prevalence

Results of the multivariable logistic regression model showed that traditional rural housing was positively associated with higher tick prevalence and the odds of acquiring higher tick prevalence on farms with traditional housing type was almost 13 times as high as the farms with open housing system. In the traditional rural housing, a farm has an uncovered and covered area. The latter consists of almost completely closed animal shed without proper ventilation and a simple roof structure and are used for protection from cold weather during the winter season, while the roof structure along with trees is used for protection during the summer and the monsoon season. The walls of the buildings are made of hard or soft bricks with mud as a seal, whereas the roof is made of bricks placed on wood or iron rods with a thin layer of mud on top. A previous study has shown that the animals were more prone to tick infestation in a closed-type of housing, which is quite similar to the traditional housing system, as compared to open-type housing [13]. It is hypothesised that less exposure to sunlight favours the retention of humidity in heaps of dung cakes and stacks of bricks in the closed houses, providing favourable sheltering places for ticks throughout the year [62]. In addition, female ticks lay eggs in cracks and crevices in the walls of animal sheds, which not only provides an optimal environment for tick development but are also a preferred hiding spot for *Hyalomma* nymphs and adults [63]. Caulking of the walls of the animal sheds is an inexpensive measure that can significantly reduce the tick burden [63].

We found that farms, where grazing was practised, had a higher tick prevalence as compared to farms with stall feeding system (OR = 12.6), which is likely to be caused by a decreased exposure to questing ticks in stall-fed animals compared to animals in pastures [13, 40]. A negative association between zero-grazing and TBDs (babesiosis and theileriosis) has also been recorded [64].

Although the use of acaricides was significantly associated (OR = 7.5) with tick prevalence and the farms, where acaricides were used, had a low tick prevalence, ticks were found on all livestock farms, suggesting that acaricide resistance might occur. So far, no study from Pakistan evaluated acaricidal resistance, but it has been reported from other countries [65]. Application of acaricides on farms has been reported the most widely used method of tick control in dairy farming [63]. A few farmers also reported the use of “Taramira”, seeds of aragula (*Eruca sativa*), to control tick infestation. In Punjab Province, Taramira is used in different ways against ticks, e.g. as “Taramira oil” topically on the animal body, whereas others prepare a mixture with salt and water and drench tick-infested animals. This formula is not only believed to decrease tick burdens, but it is also considered as a milk booster. However, there is no published report confirming the acaricidal activity of Taramira oil.

Another important risk factor associated with higher tick prevalence on the farm was the absence of rural poultry. Conversely, the farms that reared rural poultry by integrated farming with ruminants had a significantly lower tick prevalence. The OR suggested that the chances of having higher tick prevalence on farms, where rural poultry was absent, were approximately four times as high as on farms where poultry was reared. Rearing chicken on livestock farms reduces tick burden as chickens act as a natural pest control by picking ticks from animal bodies as well as from their surroundings.

In our study, flooring showed a statistically significant association with high tick infestation in univariable analysis and the farms with soft floors had a higher tick prevalence, but it appeared insignificant when included in the multivariable analysis when confounding effects were accounted for. Previous studies from Pakistan reported non-cemented (soft and mixed type) flooring as a risk factor, and the chances of being infested with ticks were two times as high as in animals kept on cemented floors [13].

## Conclusion

The most common tick species infesting ruminants in Punjab, Pakistan were *H. anatolicum* and *R. microplus. Hyalomma dromedarii* and *R. turanicus* were also found, whereby the latter species had not been reported from the study area before. Animal species, gender, age and breed were important host characteristics that determined the intensity of tick infestation. Several risk factors were found to be associated with higher tick prevalence on livestock farms, including the absence of rural poultry, not performing acaricide treatments, traditional rural housing systems and grazing. The outcomes of this study will be useful in the planning of integrated control strategies for ticks and TBDs in Pakistan.

## Additional files

Additional file 1: Table S1. Weather data for Punjab as recorded by the Pakistan Meteorological Department. (DOCX 14 kb)

Additional file 2: Table S2. Survey of livestock farms in Punjab Province (2013): Summary of categorical variables included in the questionnaire. **Table S3. **Survey of livestock farms in Punjab Province (2013): Summary of numeric variables included in the questionnaire. (DOCX 17 kb)

Additional file 3: Table S4. Survey of livestock farms in Punjab Province (2013): Summary of variables included initially in the multivariable logistic regression model. (DOCX 21 kb)

## Abbreviations

AI: Aridity index
AIC: Akaike information criterion
BLAST: Basic local alignment search tool
CCHF: Crimean-Congo haemorrhagic fever
CI: Confidence interval
*cox*1: Cytochrome *c* oxidase subunit 1
DNA: Deoxyribonucleic acid
ITS2: Second internal transcribed spacer
OR: Odds ratio
PCR: Polymerase chain reaction
TBDs: Tick-borne diseases
