# Supplementary material for: Distribution of ticks infesting ruminants and risk factors associated with high tick prevalence in livestock farms in the semi-arid and arid agro-ecological zones of Pakistan
Source: Parasit Vectors. 2017 Apr 19;10:190. doi: 10.1186/s13071-017-2138-0 (PMC5395890; doi:10.1186/s13071-017-2138-0)
Supplement: Supplementary file 1 — Weather data for Punjab as recorded by the Pakistan Meteorological Department. (DOCX 14 kb) [file 13071_2017_2138_MOESM1_ESM.docx]

**Additional file 1: Table S1.** Weather data for Punjab as recorded by the Pakistan Meteorological Department

| **Months** | **Semi-arid zone (altitude: 188-404 m)** | | | | **Arid zone (altitude: 67-180 m)** | | | |
| --- | --- | --- | --- | --- | --- | --- | --- | --- |
|  | **Mean daily temperature (°C)** | | **Rainfall (mm)** | **Relative humidity (%)** | **Mean daily temperature (°C)** | | **Rainfall (mm)** | **Relative humidity (%)** |
|  | **Max±SD** | **Min±SD** |  | **Mean±SD** | **Max±SD** | **Min±SD** |  | **Mean±SD** |
| Aug 2013 | 30.5±3 | 22.5±1.8 | 340 | 79.0±13.1 | 37.2±2.5 | 27.5±1.4 | 51 | 68.6±10.7 |
| Sep 2013 | 32.2±1.5 | 21.6±1.7 | 27.2 | 70.5±11.4 | 37.6±1.6 | 25.6±1.1 | 0 | 60.9±6.1 |
| Oct 2013 | 29.6±1.9 | 17.9±3.1 | 19.1 | 68.2±12.3 | 35.4±2.3 | 21.9±3.5 | 1 | 60.7±7.2 |
| Nov 2013 | 23.8±1.7 | 9.6±2 | 6.5 | 67.9±10.4 | 28.3±2.3 | 12.8±1.5 | 1 | 64.1±8.1 |
| Dec 2013 | 18.6±4 | 4.8±2.2 | 5.4 | 71.4±14.0 | 22.9±4.6 | 8.3±3 | 0 | 67±11.4 |
| **Average** | 26.9±2.4 | 15.3±2.2 | 79.6 | 71.4±12.2 | 32.3±2.7 | 19.2±2.1 | 10.6 | 64.3±8.7 |
