# Supplementary material for: Distribution of ticks infesting ruminants and risk factors associated with high tick prevalence in livestock farms in the semi-arid and arid agro-ecological zones of Pakistan
Source: Parasit Vectors. 2017 Apr 19;10:190. doi: 10.1186/s13071-017-2138-0 (PMC5395890; doi:10.1186/s13071-017-2138-0)
Supplement: Supplementary file 2 — Survey of livestock farms in Punjab Province (2013): Summary of categorical variables included in the questionnaire. Table S3. Survey of livestock farms in Punjab Province (2013): Summary of numeric variables included in the questionnaire. (DOCX 17 kb) [file 13071_2017_2138_MOESM2_ESM.docx]

**Additional file 2: Table S2.** Survey of livestock farms in Punjab province (2013): Summary of categorical variables included in the questionnaire

| **Variable** | **Response categories** | **No. of responses*^a^***  ***n* = 108**  **(95% CI)** | ***P* value*^b^*** |
| --- | --- | --- | --- |
| **Farm-related variables** | | | |
| Farm type | Traditional  Semi-commercial | 87.0 (79.2-92.7)  13.0 (7.3-20.8) | 0.601 |
| Rural poultry | Present  Absent | 40.7 (31.4-50.6)  59.3 (49.4-68.6) | < 0.001 |
| Dogs | Present  Absent | 36.1 (27.1-45.9)  63.9 (54.1-72.9) | 0.231 |
| Purpose | Additional source of income  Main source of income  Own supply | 69.4 (59.8-77.9)  25.0 (17.2-34.3)  5.6 (2.1-11.7) | 0.0578  0.191 |
| Ruminant species | Single ruminant species  Multiple ruminant species | 11.1 (5.9-18.6)  88.9 (81.4-94.1) | 0.454 |
| **Tick control management** | | | |
| Use of acaricide/s | Yes  No | 29.0 (20.6-38.5)  71.0 (61.5-79.4) | < 0.001 |
| Animal species treated | All the ruminant species  Only large ruminants | 19.4 (7.5-37.5)  80.6 (62.5-92.5) | 0.995 |
| Method of application | Injection  Topical  Injection and Topical | 38.7 (21.8-57.8)  41.9 (24.5-60.9)  19.4 (7.5-37.5) | 0.995  0.995 |
| Frequency of application | Once a year  2-3 times a year  Don’t know | 38.7 (21.8-57.8)  38.7 (21.8-57.8)  22.6 (9.6-41.1) | 0.995 |
| Conventional methods | Do something  Nothing | 81.6 (71.0-89.5)  18.4 (10.5-29.0) | 0.577 |
| **Housing** | | | |
| Housing type | Open  Traditional rural | 15.7 (9.4-24.0)  84.3 (76.0-90.6) | 0.002 |
| Housing material | Hard bricks with wood/iron  Soft bricks with wood/straw  Soft and Hard bricks | 73.1 (63.8-81.2)  16.7 (10.2-25.1)  10.2 (5.2-17.5) | 0.931  0.260 |
| Floor type | Soft  Hard | 52.8 (42.9-62.5)  47.2 (37.7-57.1) | 0.033 |
| Feeding method | Stall feeding – zero-grazing  Grazing | 75.9 (66.7-83.6)  24.1 (16.4-33.3) | 0.002 |
| Feed storage | Yes  No | 57.4 (47.5-66.9)  42.6 (33.1-52.5) | 0.243 |
| Boundary wall | Yes  No/Incomplete | 70.4 (60.8-78.8)  29.6 (21.2-39.2) | 0.975 |
| Trees | Present  Absent | 89.8 (82.5-94.8)  10.2 (5.2-17.5) | 0.261 |
| Frequency of removal of animal dung | Daily basis  After a long time (monthly basis) | 43.5 (34.0-53.4)  56.5 (46.6-66.0) | 0.031 |

***^a^***No. of responses = $\frac{Number of responses for specific category}{Total number of respondents} X 100$

*^b^*Probability values of univariable analysis using Fishers’ exact test

**Additional file 2: Table S3.** Survey of livestock farms in Punjab province (2013): Summary of numeric variables included in the questionnaire

| **Variable** | **Median (Q1-Q3)** | ***P* value** |
| --- | --- | --- |
| Farm size in hectares | 0.05 (0.02-0.05) | 0.756 |
| Herd size | 10.0 (8-15) | 0.846 |
| Distance to nearest livestock farm | 200 (50-400) | 0.087 |
