# Supplementary material for: Distribution of ticks infesting ruminants and risk factors associated with high tick prevalence in livestock farms in the semi-arid and arid agro-ecological zones of Pakistan
Source: Parasit Vectors. 2017 Apr 19;10:190. doi: 10.1186/s13071-017-2138-0 (PMC5395890; doi:10.1186/s13071-017-2138-0)
Supplement: Supplementary file 3 — Survey of livestock farms in Punjab Province (2013): Summary of variables included initially in the multivariable logistic regression model. (DOCX 21 kb) [file 13071_2017_2138_MOESM3_ESM.docx]

**Additional file 3: Table S4.** Survey of livestock farms in Punjab province (2013): Summary of variables included initially in the multivariable logistic regression model

| **Variable** | **Response categories** | **Odds ratio** | **95% CI** | ***P* value** |
| --- | --- | --- | --- | --- |
| Rural poultry | Present | 1 |  |  |
|  | Absent | 4.5 | 1.5-14.1 | 0.008 |
| Purpose of farming | Main source of income | 1 |  |  |
|  | Additional source of income | 1.5 | 0.5-4.6 | 0.483 |
| Use of acaricide/s | Yes | 1 |  |  |
|  | No | 7.6 | 2.3-29.7 | 0.001 |
| Housing type | Open | 1 |  |  |
|  | Traditional rural | 12.4 | 2.1-121.6 | 0.012 |
| Floor type | Soft | 1 |  |  |
|  | Hard | 1.0 | 0.3-3.2 | 0.958 |
| Feeding method | Stall feeding | 1 |  |  |
|  | Grazing | 12.8 | 2.9-98.8 | 0.003 |
| Frequency of removal of animal dung | After a long time (monthly basis) | 1 |  |  |
|  | Daily basis | 0.7 | 0.2-2.4 | 0.592 |
| Distance to nearest livestock farm | > 219 m | 1 |  |  |
|  | < 219 m | 0.7 | 0.2-2.1 | 0.513 |
